# Opioid-free anesthesia for patients with joint hypermobility syndrome undergoing craneo-cervical fixation: a case-series study focused on anti-hyperalgesic approach

**DOI:** 10.1186/s13023-021-01795-4

**Published:** 2021-04-13

**Authors:** Carlos Ramírez-Paesano, Albert Juanola Galceran, Claudia Rodiera Clarens, Vicenҫ Gilete García, Bartolomé Oliver Abadal, Verónica Vilchez Cobo, Bibiana Ros Nebot, Sara Julián González, Lucía Cao López, Jesús Santaliestra Fierro, Josep Rodiera Olivé

**Affiliations:** 1 Servei Central D’Anestesiología (Anestalia), Centro Médico Teknon, Carrer Vilana 12, 08022 Barcelona, Spain; 2 Hospital Universitari Dexeus, Grupo Quironsalud, Carrer Sabino Arana, 5, 19, 08028 Barcelona, Spain; 3 Centro Médico Teknon, Grupo Quironsalud, Carrer Vilana 12, 08022 Barcelona, Spain; 4 Postoperative Pain Management Team of Servei Central D’Anestesiología (Anestalia), Centro Médico Teknon, Grupo Quironsalud, Carrer Vilana 12, 08022 Barcelona, Spain

**Keywords:** Opiod-free-anesthesia, Craneocervical instability, Craneocervical fixation, Occipitocervical fixation, Ehlers-Danlos syndrome-hypermobility type, Joint hypermobility syndrome, Opiod-induced hyperalgesia, Central sensitization phenomena, Anti-hyperalgesic infusions

## Abstract

**Background:**

Patients with Ehlers-Danlos Syndrome/Hypermobility Type (EDS-HT/JHS) and Craneo-Cervical Instability frequently suffer from severe widespread pain which is difficult to control. Chronic neuroinflammation, opioid-induced hyperalgesia, and central sensitization may explain this painful condition. The aim of this study was to determine if opioid-free anesthesia plus the postoperative administration of lidocaine, ketamine and dexmedetomidine can reduce postoperative pain and the need of methadone rescues in comparison with opioid-based management in these patients undergoing Craneo-Cervical Fixation (CCF). The secondary aim was to assess the needs of opioids at hospital-discharge, incidence of gastrointestinal complications and the requirement of anxiolytic.

**Methods:**

A retrospective, consecutive case series study was designed. 42 patients with EDS-HT/JHS undergoing CCF were enrolled in two groups: an OFA-plus Group that received opioid-free anesthesia with propofol, lidocaine, ketamine and dexmedetomidine, and OP Group, opioid-based anesthesia-analgesia. The main variables: Preoperative Visual Analogue Score (VAS), postoperative VAS on the 1st, 2nd, 4th and 6th days, sufentanil or morphine requirements, need for methadone rescue, and VAS at hospital-discharge. Data was presented by mean ± SD, percentage, median or interquartile range. Chi-squared or Fisher’s test. 95% C.I and *P* values < 0.05.

**Results:**

Nineteen patients in OFA-plus, and 23 patients in OP group. VAS was lower in OFA-plus on the postoperative days evaluated (*p* < 0.001).VAS at hospital-discharge was lower in OFA-plus: 4.96 (4.54–5.37) vs. OP 6.39 (6.07–6.71) (*p* < 0.001). Methadone requirement was lower in the OFA-plus (*p* < 0.001). 78% of patients in OFA-plus didn’t need methadone rescue. 95% in OP group needed methadone rescues at high doses(> 15 mg/day). No differences regarding equivalent doses of sufentanil or morphine consumption on the 2nd, 4th, and 6th postoperative days were found. OFA-plus decreased ileus, nausea and vomiting (*p* < 0.001). 60.9% in OFA-plus group decreased opioid requirements at hospital-discharge compared with preoperative values. A 77% reduction of anxiolytics requirements was shown.

**Conclusion:**

OFA-plus management for patients undergoing CCF with EDS-HT/JHS shows significant reduction in postoperative pain and at hospital-discharge compared with opioid-based anesthesia. OFA-plus management decreases the total doses of methadone rescues, reduces anxiolytic requirements and gastrointestinal side-effects, except for constipation. OFA-plus management is a feasible option to improve postoperative pain control, reducing the opioids’ use and their postoperative side-effects in patients undergoing CCF with EDS-HT/JHS.

## Introduction

Cranio-cervical instability (CCI) has been well identified in diseases with connective tissue disorders like Ehlers-Danlos Syndrome-Hypermobility Type/Joint Hypermobility Syndrome (EDS-HT/JHS). Generalized joint hypermobility can exhibit laxity of the ligaments of the spine and a propensity to have severe symptoms due to CCI and Cervical Medullary Syndrome (CMS) that may be involved in the development of severe proprioceptive disturbances causing soft tissue microtrauma and generalized musculoskeletal pain [1, 2]**.**

Furthermore, CMS may explain some of the neurological and ancillary symptoms of the patient with CCI and JHS. An unstable cervical spine may cause functional brainstem compression that is possibly influenced by neck movements and axonal damage due to deformative stress [3]. Defined as a group of bulbar symptoms and myelopathy, CMS has been well described in a recent Consensus Statement on Craniovertebral instability [4]. CMS may be explained by the traumatic deformation of axons that induces abnormal sodium influx through mechanically sensitive Na^+^ channels. This subsequently triggers an increase in intra-axonal calcium via the opening of the voltage-gated calcium channel, up-regulation of glutaminergic pathway,chronic neuro-inflammation and apoptosis.

CMS symptoms include altered vision (particularly photophobia, diplopia), altered hearing (peculiar misophonia), altered speech and swallowing, the presence of vertigo, dizziness, numbness (i.e., peripheral hypo/anesthesia), dysesthesias (e.g., allodynia, hyperalgesia, burning sensations, etc.),paresthesia, tremulous limbs, muscle weakness, lack of balance and coordination, abnormal movements (e.g., fasciculations, periodic limb movements, dystonia), sensory loss, bladder dysfunction,altered sleep architecture, mood changes, emotional and cognitive disturbances(minor memory and concentration disturbances) [5]. Some of these symptoms coincide with those observed in chronic fatigue syndrome (CFS), Myalgic Encephalomyelitis (ME),or a combination of both (ME/CFS) [6].

CCI may give rise to microtraumas on cranio-cervical joints surfaces and musculoskeletal inflammation with repetitive peripheral sensitization that ultimately result in the complex phenomenon of Central Sensitization(CS) and hyperalgesia [2].

Patients with EDS-HT/JHS and CCI suffer from severe widespread pain. This pain is exceedingly difficult to manage and is often poorly controlled with opioids. Moreover, these patients have frequently associated chronic fatigue, high level of anxiety, depression or mood changes, functional gastrointestinal disturbances, mast cell activation syndrome (MCAS), and autonomic symptoms such as postural orthostatic tachycardia syndrome (POTS). On the other hand, it is striking that some studies seem to suggest there is a common genetic condition related to germline excessive duplications and triplications in the allelic TPSAB1 gene encoding α-tryptase that provokes an increase in serum basal tryptase levels from mast cell activity (tryptase ≥ 8.0 ng/ml). This is a common autosomal dominant inheritance that may partially explain an important intolerance of opioids, the coexistence of these multi‐system symptoms affecting the skin, gastrointestinal and urinary tract, circulation and musculoskeletal system as well as for the coexistence of MCAS, POTS and EDS-HT/JHS [7, 8].

Opioid-induced hyperalgesia (OIH), chronic neuro-inflammation, glial activation and neuronal plasticity in the spinal cord, brainstem and brain, and consequently Central Sensitization (CS) phenomena may explain this complex and painful condition [1, 9–11].

In fact, these patients may suffer from a category of pain known as central intractable pain, a painful condition that does not respond well to opioids and their use may even be detrimental to the patient [11, 12].

Enhanced Recovery After Surgery (ERAS) protocols have incorporated some opioid-free anesthetic techniques (OFA) thanks to the use of infusions of coadjuvant drugs with anti-hyperalgesic and analgesic properties with mechanisms of action different to opioids. The medical literature supports the use of intravenous infusions of lidocaine, ketamine and dexmedetomidine as a balanced way to substitute or reduce opioids in the perioperative period [11–15].

Considering that OIH and CS are important causes of this complex and painful condition, the restriction of the use of opioids and the use of drugs with potent anti-hyperalgesic effects seems to be a reasonable option for the management of perioperative pain in patients with EDS-HT/JHS and cranio-cervical instability undergoing cranio-cervical fixation/fusion surgery (CCF) [13–16].

The main aim of this study was to determine if the use of OFA plus the postoperative infusions of low intravenous doses of lidocaine, ketamine and dexmedetomidine (OFA-plus) protocol improves postoperative pain control, and reduces the need for opioid rescues (methadone) compared to those patients managed with opioid-based anesthesia and analgesia. The secondary aim was to determine if the restricted use of opioids protocol may reduce the need for opioids at hospital discharge, decrease the postoperative incidence of gastrointestinal complications (nausea, vomiting, ileus, constipation) and reduces the need for postoperative anxiolytic treatment.

## Methods

An observational, retrospective and descriptive case-series study was conducted at “Centro MédicoTeknon” (Quirónsalud Group) after approval by the ethical committee of the hospital (2020/107-ANE-CMT), and registered in Clinical Trials (ClinicalTrials.gov ID: NCT04437589). Forty-two patients, thirty-nine women and three men diagnosed with EDS-HT/JHS undergoing CCF from September 2018 to March 2020 were enrolled for this study. Inclusion criteria were men and women from 18 to 60 years old with a diagnosis of EDS-HT/JHS who underwent CCF. Patients were divided into two groups: the OFA-plus Group, patients who received opioid free total intravenous anesthesia with propofol, lidocaine, ketamine and dexmedetomidine, and the OP Group, opioid-based total intravenous anesthesia with propofol. Exclusion criteria were CCF due to post-traumatic or oncologic cranio-cervical instability, lidocaine allergy, advanced heart blockage, non-medicated epilepsy or convulsive syndrome. Informed consent was signed by all participants.

### OFA-plus group

**Fig. 1:**
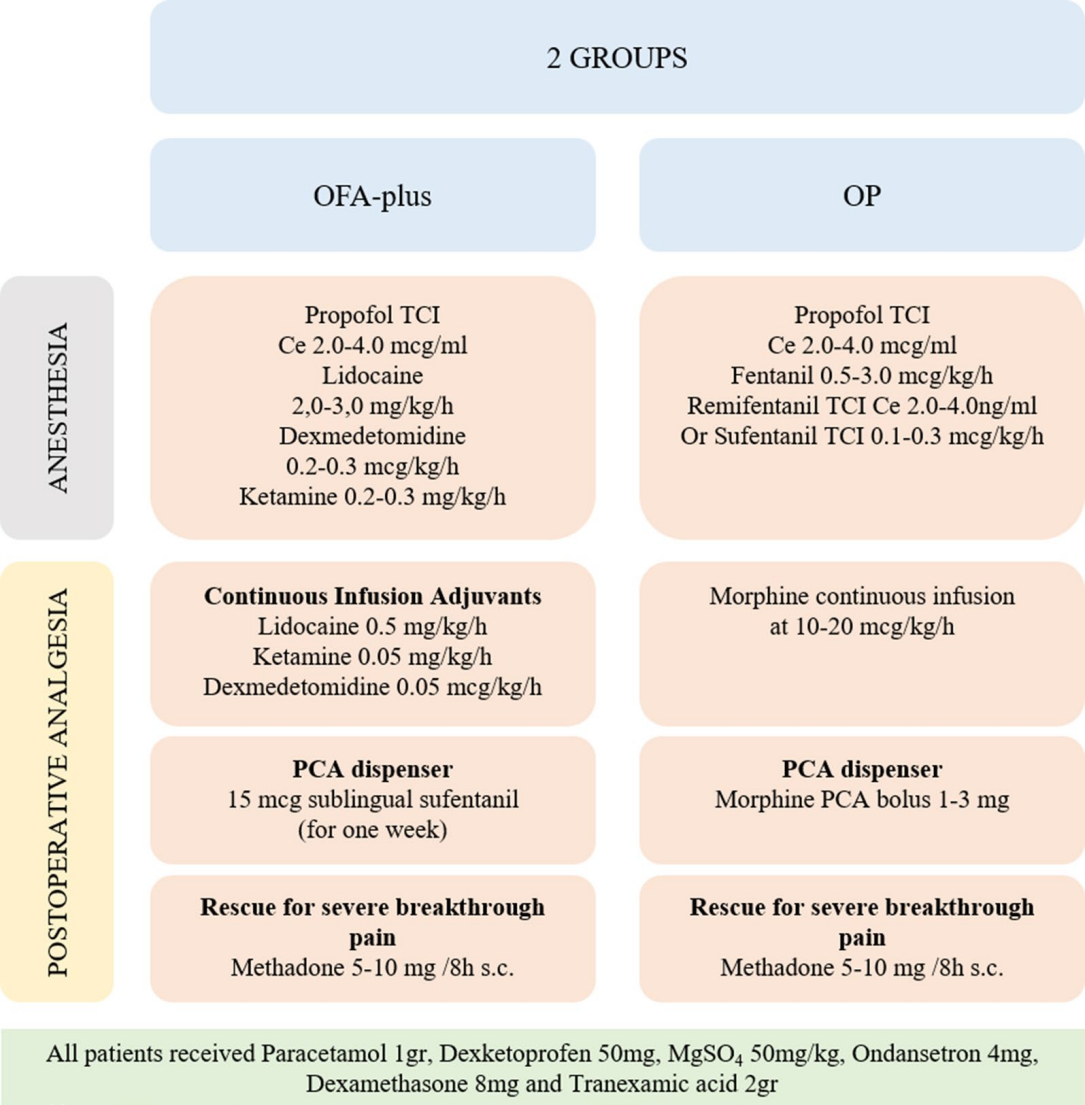
Anesthesia and postoperative analgesia protocol according to each group

Opioid free total intravenous anesthesia consisted of propofol infusion by Braun Infusomat®Space pump with Target Controlled Infusion mode (TCI) Schnider model at Ce. 2.0–4.0 mcg/ml to maintain BIS values between 40–60, lidocaine 2.0–3.0 mg/kg/h, ketamine 0.2–0.3 mg/kg/h and dexmedetomidine 0.2–0.3 mcg/kg/h. Postoperative multimodal analgesia was based on lidocaine 0.5 mg/kg/h, ketamine 0.05 mg/kg/h and dexmedetomidine 0.05 mcg/kg/h infusions for a week. For breakthrough severe pain crisis 15 mcg of sufentanil (sublingual micro-pills) was administered by Patient Controlled Analgesia (PCA) system with 20 min lock-out (Zalviso™: PCA dispenser), and 5–10 mg of subcutaneous methadone if there was no improvement despite previous measures. In *the OP Group*, total intravenous anesthesia consisted of propofol by TCI mode Schnider model, fentanyl 0.5–3.0 mcg/kg/h and remifentanil infusion by Braun Infusomat®Space pump TCI Minto model at Ce 2.0–4.0 ng/ml, or sufentanil 0.1–0.3 mcg/kg/h. Postoperative analgesia with morphine infusion at 10–20 mcg/kg/h plus PCA bolus 10–30 mcg/kg/h 20 min lock-out. Additionally, 5–10 mg subcutaneous methadone for breakthrough severe pain crisis was administered if no improvement was shown despite previous measures. (Fig. 1).

Main and secondary variables were collected according Table 1.

**Table 1:** Description of main and secondary variables

*Main variables*
Preoperative VAS score (0–10/10). 0 means absence of pain and 10 the severest pain imaginable
Postoperative VASscore (0–10/10) at 1st, 2nd, 4th, 6th postoperative days and at hospital discharge
^(*)^Range of sufentanil requirements (micro-pills per postoperative day)
1: 0–2 micro-pills (equal or less than 30 mcg/day)
2: 2–4 micro-pills (30–60 mcg/day)
3: 5–10 micro-pills (75–150 mcg/day)
4: More than 10 micro-pills (more than 150 mg/day)
^(*)^ Range of morphine requirements per day (continuous perfusion plus PCA)
1: 0–30 mg/day
2: 30–60 mg/day
3: 60–150 mg/day
4: More than 150 mg/day
Requirement of methadone for breakthrough severe pain crisis
0: no use
1: < 10 mg/day
2: 10–15 mg/day
3: More than 15 mg/day
*Secondary variables*
Length of hospital stay (days)
Preoperative opioid requirement
0: No opioids or occasionally
1: Weak opioids (tramadol, codeine, tapentadol)
2: Strong opioids (fentanyl, oxycodone, morphine, buprenorphine)
3: Strong opioid combination
Opioid requirement at discharge
0: No opioids
1: Decrease compared to preoperative (between 20 and 30%)
2: Same dose compared to preoperative
3: More dose compared to preoperative
Postoperative nausea and vomiting
1: Yes
2: No
Paralytic ileus
1: Yes
2: No
Constipation
1: Yes
2: No
Requirement of Anxiolytics
0: No requirements
1: Once/day
2: Twice/day
3: 3 Times/day
4: More than 3 times/day

*VAS* visual analogue scale score

^(*)^Opioid equivalences:

^(*)^In order to compare daily and total opioid requirements (morphine vs. sufentanil), the following equivalence was assumed according to equipotent doses:

15 mg morphine (intravenous) = 15 mcg sufentanil (Sublingual) = 150 mcg sublingual fentanil

Based on the following equivalences:

10 mg i.v morphine = 100 mcg sublingual fentanyl = 30 mg oral morphine

Fentanil: sufentanil 10:1

### Statistical analysis

Information was extracted from the database of postoperative analgesia team of Anesthesiology Department (Servei Central d' anesthesia-Anestalia) and from the hospital medical records in “Centro MédicoTeknon”. An identification was assigned by a numerical code to every patient in order to preserve the patient’s privacy. Descriptive statistics were expressed by mean ± standard deviation. Categorial variables were presented as frequency (percentages). Depending on the variables Chi-squared or Fisher’s test were used for inferential statistics. Non- normally distributed data were shown as median and 25th percentile-75th percentile (IQR: Q1–Q3). Confidence interval of 95% (C.I), and *P* values < 0.05 were considered to be significant.

## Results

19 patients in OFA-plus group and 23 patients in OP group were analyzed. No significant differences were found in BMI in OFA-plus 23.5 ± 4.30 kg/mt^2^ vs. OP 22.9 ± 4.43 kg/mt^2^. There were also no differences in preoperative VAS between both groups 7.57 (6.99–7.96) and 7.47 (7.12–8.01), respectively.

**Fig. 2:**
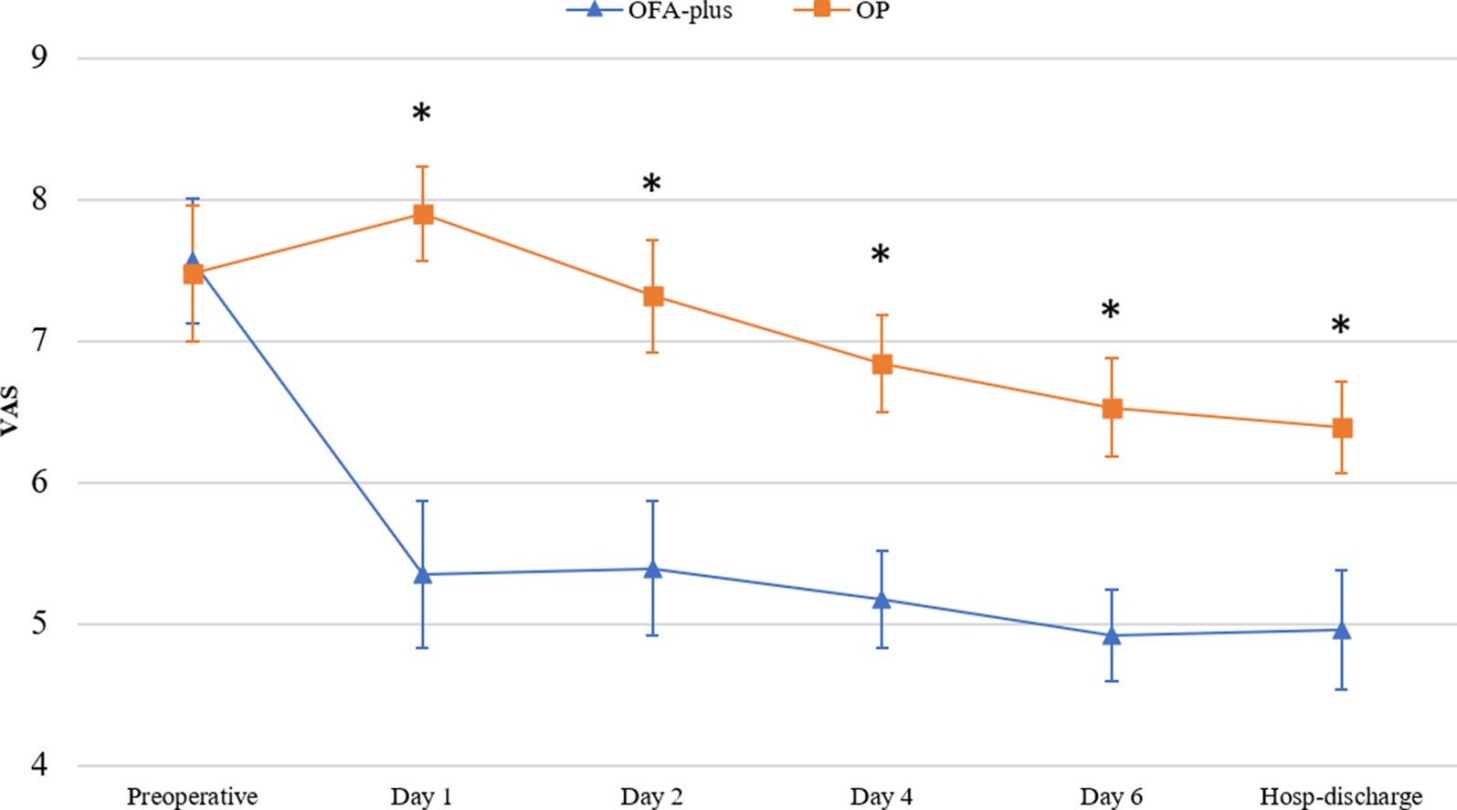
Comparison of perioperative visual analogue scale (VAS) scores at rest. * (*p* < 0.01)

There was a reduction in VAS scores during 1st, 2nd, 4th, 6th postoperative days in OFA-plus group (*p* < 0.001). The reduction of VAS was more important on the 1stpostoperative dayin the OFA-plus group 5.35 (4.83–5.86) vs. OP group 7.89 (7.56–8.23) (*p* < 0.001), meaning a decrease up to 32% of VAS in OFA-plus group. A reduction of VAS scores at hospital discharge day in OFA-plus group 4.96 (4.54–5.37) was also found in comparison with the OP group 6.39 (6.07–6.71) (*p* < 0.001) (Fig. 2). For statistical analysis at hospital discharge day one patient of the OP group was excluded due to death because of respiratory depression. There were no differences in the length of the hospital stay (OFA-plus: 19 ± 3.1 days vs. OP 22 ± 2.3 days).

**Fig. 3:**
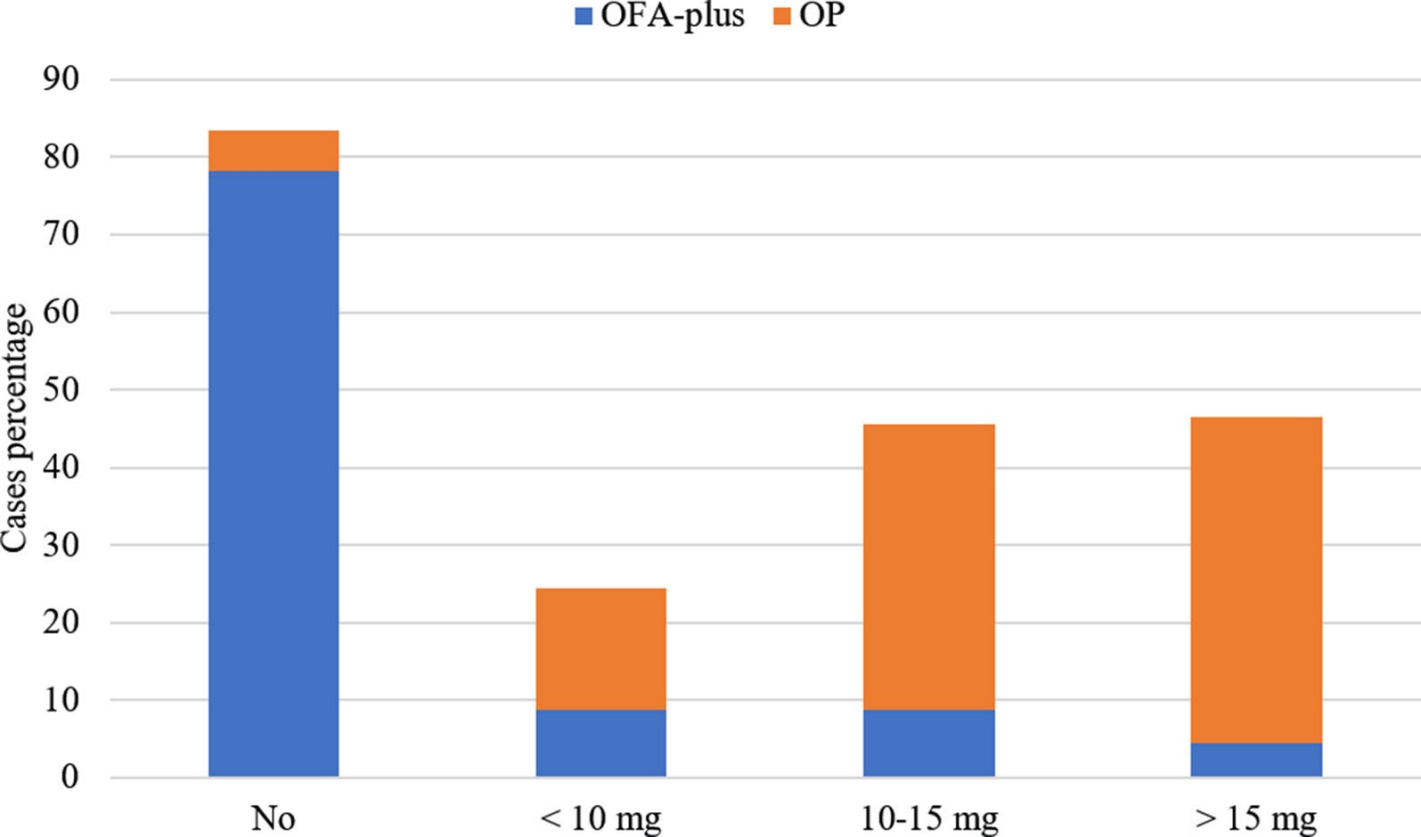
Percentual distribution of patients that needed methadone rescue during hospitalization. * p < 0.01

There were differences regarding the use of methadone to treat severe breakthrough pain crisis. Up to 78% of patients in OFA-plus group did not need methadone rescue. 95% of patients in the OP group needed methadone rescue. About 42% of OP patients required high doses of methadone (more than 15 mg/day) (Fig. 3).

There was a significant reduction of sufentanil requirement in the OFA-plus group during the 1st postoperative day in comparison with the equivalent doses of morphine consumption in the OP group (*p* < 0.05) There were no differences regarding equivalent doses of sufentanil or morphine consumption on 2nd, 4th, and 6th postoperative days between both groups (Table 2). The period of administration of sufentanil by PCA was 7.0 ± 2.2 days.

**Table 2:** Perioperative VAS score and opioid requirements

Variable	Total (n = 42)	Opioid-free (n = 23)	Opioid (n = 19)	P value
(*) VAS score, (median, Q1–Q3)				
Preoperative	8 (7–8)	8 (7–8)	7 (7–8)	0.764
Day 1	6.50 (5–8)	5 (5–6)	8 (8–8)	< 0.001
Day 2	6 (5–8)	5 (5–6)	8 (7–8)	< 0.001
Day 4	6 (5–7)	5 (5–5)	7 (7–8)	< 0.001
Day 6	5 (5–7)	5 (5–5)	7 (6–7)	< 0.001
Hospital discharge day	6 (5–6.5)	5 (4–5)	6.5 (6–7)	< 0.001
Methadone rescue, n (%)				
0	19 (45.2)	18 (78.3)	1 (5.3)	< 0.001
5 mg/day	5 (11.9)	2 (8.7)	3 (15.8)	
10–15 mg/day	9 (21.4)	2 (8.7)	7 (36.8)	
> 15 mg/day	9 (21.4)	1 (4.3)	8 (42.8)	
Sufentanil (S) vs. morphine (M) on 1st, 2nd, 4th and 6th postoperative days				
Day 1, n (%)				
(S) ≤ 30 mcg/day or (M) ≤ 30 mg/day	10 (23.8)	3 (13)	7 (36.8)	0.027
(S)30–60 mcg/day or (M)30–60 mg/day	7 (16.7)	7 (30.4)	0 (0)	
(S)60–150 mcg/day or (M)60–150 mg/day	11 (26.2)	7 (30.4)	4 (21)	
(S) > 50 mcg/day or (M) > 50 mg/day	14 (33.3)	6 (26.1)	8 (42.1)	
Day 2, n (%)				
(S) ≤ 30 mcg/day or (M) ≤ 30 mg/day	11 (26.2)	3 (13)	8 (42.1)	0.201
(S)30–60 mcg/day or (M)30–60 mg/day	9 (21.4)	7 (30.4)	2 (10.5)	
(S)60–150 mcg/day or (M)60–150 mg/day	15 (35.7)	9 (39.1)	6 (31.6)	
(S) > 50 mcg/day or (M) > 50 mg/day	7 (16.7)	4 (17.1)	3 (15.8)	
Day 4, n (%)				
(S) ≤ 30 mcg/day or (M) ≤ 30 mg/day	11 (26.2)	3 (13)	8 (42.1)	0.109
(S)30–60 mcg/day or (M)30–60 mg/day	13 (31)	10 (43.5)	3 (15.8)	
(S)60–150 mcg/day or (M)60–150 mg/day	12 (28.6)	7 (30.4)	5 (26.3)	
(S) > 50 mcg/day or (M) > 50 mg/day	6 (14.3)	3 (13)	3 (15.8)	
Day 6, n (%)				
(S) ≤ 30 mcg/day or (M) ≤ 30 mg/day	10 (23.8)	3 (13)	7 (36.8)	0.224
(S)30–60 mcg/day or (M)30–60 mg/day	22 (52.4)	15 (65.2)	7 (36.8)	
(S)60–150 mcg/day or (M)60–150 mg/day	8 (19)	4 (17.4)	4 (21)	
(S) > 50 mcg/day or (M) > 50 mg/day	2 (4.8)	1 (4.3)	1 (5.3)	

*VAS* Visual Analogue Scale score, *S* sufentanil, *M* morphine, *Q1* quartile 1, *Q3* quartile 3

Data are presented as median (quartile 1, quartile 3), or n (%) depending on the type and distribution. Values < 0.05 are considered to represent a negligible difference

A reduction of ileus and postoperative nausea and vomiting was observed in the OFA-plus group (*p* < 0.001). There was no difference in constipation incidence between groups. The general consumption of anxiolytics in the OFA-plus group during the hospitalization was reduced. A 77% reduction of anxiolytics requirements was observed in OFA-plus group.

63% of patients of the OP group needed anxiolytics very frequently (more than 3 times/day). In the OFA-plus group 17.4% (n = 4) of patients had visual hallucinations. Haloperidol was used in two patients (Table 3).

**Table 3:** Demographics and secondary variables

Variable	Total (n = 42)	OFA-plus (n = 23)	OP (n = 19)	*P* value
Age (y), (means ± SD)	34.83 ± 11.06	34.04 ± 11.39	35.79 ± 10.77	0.820
Sex, n (%)				
Male	3 (7.1)	0 (0)	3 (15.8)	0.084
Female	39 (92.9)	23 (100)	16 (84.2)	
Preoperative opioid prescription, n (%)				
No or eventually	7 (16.7)	3 (13)	4 (21.1)	0.819
Weak opioids	11 (26.2)	6 (26.1)	5 (26.3)	
Strong opioids	15 (35.7)	8 (34.8)	7 (36.8)	
Strong opioid combination	9 (21.4)	6 (26.2)	3 (15.8)	
Ileus, n (%)	14 (33.3)	1 (4.3)	13 (68.4)	< 0.001
PONV, n (%)	23 (54.8)	6 (26.1)	17 (89.5)	< 0.001
Constipation, n (%)	14 (33.3)	6 (26.1)	8 (42.1)	0.335
Anxiolytic requirements, n (%)				
No	0 (0)	0 (0)	0 (0)	0.002
Possible	0 (0)	0 (0)	0 (0)	
Moderate	8 (19)	8 (34.8)	0 (0)	
Frequent	18 (42.9)	11 (47.8)	7 (36.8)	
Very frequent	16 (38.1)	4 (17.4)	12 (63.2)	
Opioid requirements at discharge^a^, n (%)				
No	8 (19)	6 (26.1)	2 (10.5)	< 0.001
Decrease	14 (33.3)	14 (60.9)	0 (0)	
Equal	8 (19)	3 (13)	5 (26.3)	
Increase	12 (28.6)	0 (0)	12 (63.2)	

Data are presented as means ± standard deviation, or n (%) depending on the type and distribution. *P* values < 0.05 are considered significant

*PONV* postoperative nausea and vomiting, *SD* standard deviation

^a^Compared to preoperative values

60.9% of patients in the OFA-plus group showed a decrease in opioid requirements at discharge compared with preoperative values. In the OP group no decrease was observed (Table 3).

## Discussion

The present study demonstrates that the patients with EDS-HT/JHS undergoing CCF who received OFAplus postoperative administration of lidocaine, ketamine and dexmedetomidine infusions presented a significant reduction in pain during the first 6 days of postoperative and at the time of the hospital discharge compared with the group managed with opioid-based anesthesia plus postoperative analgesia with intravenous morphine. Moreover, throughout the hospital stay, the percentage of patients who required methadone rescue to treat severe breakthrough pain was considerably lower in the OFA-plus group, as well as the total doses of methadone.

Due to the limitations of a retrospective study, there were difficulties in achieving some of our goals. The patients of the OFA-plus group used oral sufentanil by PCA as part of their postoperative analgesia protocol, and patients of OP group received morphine in continuous infusion plus PCA bolus. The difficulty found in establishing a comparison in the consumption of opioids by PCA between both groups during the first 6 postoperative days was solved using the concept of equivalence potency between opioids, and also grouping the opioid requirements by the ranges of clinical doses used.

During the first postoperative day, PCA requirements of sufentanil in the OFA-plus group were lower than the equivalent doses of morphine required in the OP group. This finding is consistent with the published literature related to the effects of the OFA on the reduction of opioids in the first 24 h after surgery. Maintaining intraoperative homeostasis (normovolemia, normothermia, normoglycemia, hemodynamic stability) in conjunction with the doses of lidocaine, ketamine and dexmedetomidine used during surgery probably had a significant effect on the attenuation of intraoperative stress, systemic inflammatory response and pain on the first postoperative day [13, 17].

Although there were no differences in sufentanil or morphine equivalent requirements on the 2nd, 4th and 6th day, VAS score was significantly lower in the OFA-plus group during the first postoperative week. The complexity of the patients studied and the type of surgery made analgesic treatment without opioids extremely difficult for pain control in the first days after surgery [16, 18].

Studies of OFA in patients undergoing spinal surgery show controversial results in terms of the postoperative reduction of opioids, the recovery time, the complications, and the length of hospital stay [8–10]. However, there seems to be more agreement about the perioperative use of non-opioid coadjuvants as part of a multimodal analgesia protocol to achieve enhanced recovery after spine surgery [16, 18, 19].

We cannot affirm that OFA management is an independent factor in reducing postoperative pain. However, OFA management plus postoperative use of lidocaine, ketamine, and dexmedetomidine infusions as part of robust multimodal analgesia can explain our results [15].

Intravenous lidocaine, ketamine, and dexmedetomidine have analgesic mechanisms mediated by a strong systemic anti-inflammatory effect and other multiple anti-nociceptive pathways (i.e., reduction of inflammatory biomarkers by direct action on cell membrane of monocytes, neutrophils and mast cell, PKC-mediated reduction of Ca^++^intracellular influx and K^+^_A_-channels, action over cholinergic, adrenergic, GABAergic, NMDAr, and NK-1r pathways) [20–23].

In the OFA-plus group, these coadjuvants were administered intraoperatively and during the first postoperative week. The continued perioperative use of lidocaine, ketamine and dexmedetomidine infusions and the gradual reduction of the doses over one week might overcome the peak of the inflammatory surgical-response, and therefore its effect on pain and Central Sensitization, minimizing opioid exposure, and resulting in a reduction of VAS [12, 17, 24, 25].

In the OP group, morphine analgesia protocol was maintained for 16.0 ± 3.0 days. In the OFA-plus group, the average time of administration of sufentanil by PCA was 7.0 ± 2.2 days. During the remaining time of hospitalization of the OFA-plus group only methadone was used as a rescue pain reliever until hospital discharge. Methadone is an opioid with an unique central nervous system effects (anti-NMDA receptor and inhibition of serotonin and norepinephrine uptake) that may enhance recovery by attenuating the development of hyperalgesia and tolerance. Also, the literature describes that methadone can improve postoperative analgesia and the long-term outcome in patients undergoing posterior spinal fusion surgery [26, 27].

There was no difference in the length of the hospital stay (OFA-plus: 19 ± 3.1 days vs. OP 22 ± 2.3 days). However, at the time of hospital discharge VAS score and total opioid consumption were lower in the OFA-plus group. We believe that the results obtained are the consequence of the sum of multifactorial effects because of the administration of OFA, postoperative use of infusions of lidocaine, ketamine and dexmedetomidine, the limited use of sufentanil for less than 8 days, and the use of methadone as the only rescue during the rest of hospitalization.

Patients in both groups had preoperative severe occipital-cervical pain (VAS score OFA-plus: 8.0 vs. OP: 7.0, *p* = 0.764). Also, a high percentage of the patients in both groups (57.1%) came with medical prescription of strong opioids or a combination of these (*p* = 0.819).

At the time of hospital discharge, the OFA-plus group showed a decrease in opioid requirements in comparison to preoperative doses vs. OP group (*p* < 0.001). The 60.9% of patients in the OFA-plus group showed a reduction in opioid doses, whereas 63.2% of patients in the OP group showed an increase in comparison to preoperative requirements. In the OFA-plus group, 26.1% of patients (n = 6) were discharged without opioid prescription vs. 10.5% in the OP group.

The pain management in these patients is very complex, and it is extremely difficult not to use opioids after surgery. Although the results of our study showed a reduction in opioid requirements at the time of hospital discharge in the group with OFA-plus group, the limited number of cases studied does not allow us to give a definitive conclusion on the matter. The majority of patients who were discharged without opioid prescription had preoperative medical prescription with no opioids or weak opioids. That fact strongly suggests that opioid-induced hyperalgesia and opioid tolerance are important mechanisms that lead to the persistence of severe pain in these patients [10, 11, 28].

A high percentage of the patients undergoing CCF with EDS-HT/JHS suffer from functional gastrointestinal disturbances with tendency to develop intestinal ileus, nausea and vomiting. Also, they frequently have altered sleep architecture, mood changes, depression and emotional and cognitive disturbances. Preoperative depression or anxiety have been associated with a greater likelihood of adverse outcomes and increased opioid consumption in patients undergoing cervical spine surgery. Our results showed a significant decrease in nausea and vomiting and reduction in the use of anxiolytics in the OFA-plus group [1, 2, 29].

It seems evident that the lower exposure to opioids in the OFA-plus group contributed to a better postoperative evolution of gastrointestinal function. However, the incidence of constipation was similar in both groups, therefore the preventive use of medication to improve the quality of faeces is important. These patients remain in bed for a long time, have little mobility, a deterioration in physical condition and fluid intake is generally low.

On the other hand, the reduction in the use of anxiolytics in the OFA-plus group may be the result of low-doses of ketamine on mood, the anxiolytic effect of dexmedetomidine, and a better postoperative pain control. Almost all patients studied were young women. The effects of low-dose ketamine as an anti-depressant have been described to be better in young women and adolescent females, as well as producing lower incidence of adverse psychomimetic effects (hallucinations, anxiety or panic attacks) compared to men. In our series, 17.4% (*n* = 4, one man) of patients had visual hallucinations that justified the temporary suspension of ketamine and the subsequent dose reduction. Haloperidol was administered to two patients, improving symptoms [30].

Another limitation of our study was to evaluate the VAS only at rest. Most of these patients began to sit after 6 postoperative days, and get up or walk after a week, therefore, the VAS score during movement was not considered within our goals. An attempt was made to establish a statistical measure of inter-observers concordance by Kappa Coefficient. However, it was not possible since the recorded data of VAS evaluation was carried out and registered by a single observer, assigned daily for the assessment of pain. Both the workflow organization of our postoperative pain management team and the retrospective design of the present study represented a limitation to the definition of any inter-rater reliability coefficient [31].

Mast cell activation syndrome (MCAS) and autonomic symptoms like postural orthostatic tachycardia syndrome (POTS) are frequently present in the patients with EDS-HT/JHS [32].

Some opioids can be MCAS triggers or provoke hemodynamic disturbances due to histamine release. Moreover, many patients with EDS-HT/JHS and MCAS have a medical history of allergy to certain opioids. A thorough preoperative anamnesis should always be done to avoid using triggers [33]. Due to the aforementioned, it seems that reducing the use of opioids in these patients is reasonable. Although opioids were used in both groups throughout the hospital stay, there were no events associated with MCAS [34].

In conclusion, it is feasible to administer OFA to patients undergoing CCF with EDS-HT/JHS. Intraoperative plus postoperative use of lidocaine, ketamine, and dexmedetomidine infusions offer better postoperative pain control compared to opioid-based anesthetic/analgesic techniques. The anti-inflammatory and anti-hyperalgesic effects of lidocaine, ketamine, and dexmedetomidine may provide a beneficial effect as part of a more robust multimodal analgesia protocol focused on the management of hyperalgesia and Central Sensitization phenomena.

Although there was no evidence of a reduction in opioid use during the first postoperative week, in the OFA-plus group a lower percentage of patients required methadone throughout the hospitalization period. Moreover, OFA-plus group showed a significant reduction in VAS score compared to OP group throughout hospitalization and discharge. Furthermore, in this group there was a tendency to reduce opioid requirements at the time of hospital discharge compared to preoperative use. Unfortunately, our study did not include a long-term outcome of the patients, another aspect that limits our conclusions about the possible benefits of reducing the perioperative use of opioids in this context.

Certain doubts arise from the present study. First, what would the optimal time to maintain postoperative infusions of lidocaine, ketamine and dexmedetomidine be? Second, what oral medications could replace the effects of these infusions after one week of the postoperative period? For example, it could be lamotrigine, carbamazepine, memantine or ketamine (both anti-rNMDA), or maybe clonidine. Thirdly, could sufentanil be replaced by some other type of non-opioid analgesic rescue (i.e., medical cannabinoids)? It is probable that many other questions will arise. For example, it may be interesting to study the effect on SC and postoperative VAS of using Low-dose of Naltrexone (LDN: 1–5 mg/day) several weeks prior to surgery to reduce the chronic glial inflammation by modulating Toll-like receptor 4 signaling, and additionally by a systemic up-regulation of endogenous opioid signaling by acting on filamin A, a scaffolding protein involved in µ-opioid receptor signiling [35, 36].

## Conclusion

The present study demonstrates that the use of medications such as lidocaine, ketamine, and dexmedetomidine during the intraoperative and postoperative in patients with EDS-HT/JHS undergoing CCF is a feasible and safe option to improve postoperative pain control, and to reduce the postoperative side effects due to its anti-inflammatory, anti-hyperalgesia and opioid-sparing effects. We believe that the information derived from this retrospective study can offer an open door for future research, prospective and controlled clinical trials.

## Data Availability

Datasets used and/or analyzed during the current study are available from the corresponding author on reasonable request.

## Abbreviations

Ce: Concentration at pharmacokinetic effect compartment
OCF: Occipito-cervical fixation/fusion surgery
CCF: Cranio-cervical fixation/fusion surgery
CCI: Cranio-cervical instability
C.I.: Confidence interval
CS: Central sensitization phenomena
EDS-HT/JHS: Ehlers–Danlos syndrome-hypermobility type/joint hypermobility syndrome
ERAS: Enhanced recovery after surgery protocols
GABA: Gamma-aminobutyric acid
IQR: Interquartile range
K^+^_A_: Potassium channels type A
MCAS: Mast cell activation syndrome
NMDAr: N-Methyl-D-Aspartate receptors
NK-1r: Neurokinin-1 receptors
PCA: Patient controlled analgesia
OFA: Opioid-free anesthesia
OFA-plus: Opioid-free anesthesia plus postoperative infusions of lidocaine, ketamine and dexmedetomidine
OIH: Opioid-induced hyperalgesia
PKC: Protein Kinase C
PONV: Postoperative nausea and vomiting
OP: Opioid-based anesthesia and analgesia management
POTS: Postural orthostatic tachycardia syndrome
SD: Standard deviation
TCI: Target controlled infusion mode
VAS: Visual analogue scale score
